# Guidelines for the use of flow cytometry

**DOI:** 10.1002/iid3.207

**Published:** 2017-11-17

**Authors:** Marc Veldhoen

**Affiliations:** ^1^ Instituto de Medicina Molecular, Faculdade de Medicina da Universidade de Lisboa Av. Professor Egas Moniz Lisbon 1649‐028 Portugal

The editorial team of *European Journal of Immunology*, under the guidance of Professor Andreas Radbruch and a large international team of the immunological and flow cytometry core facilities community, have recently published the “Guidelines for the use of flow cytometry and cell sorting in immunological studies” 1. At *Immunity, Inflammation and Disease*, published in the Wiley immunology journals programme alongside *European Journal of Immunology*, we would like to fully endorse the implementation of these guidelines in the use of studies and as a guide to reporting flow cytometry based results in publications.

Analysing thousands of cells a second, determining protein, DNA, RNA, protein modification and other cell product levels, as well as cell cycle and viability status and division rate at a single cell level and, in addition, having the option to sort (live) single cells based on user determined parameters, accurately and simultaneously detecting many different protein levels in small sample volumes such as serum (cytometric bead array (CBA) assays) and providing these results repetitively, has become the norm in biomedical sciences, especially the field of immunology. Flow cytometry has developed very rapidly in the past five decades, from its first use to measure DNA to its multi‐parameter abilities and linkup with mass‐spectrometry (CyTOF) and single cell‐sequencing technologies. Although often portrayed as the workhorse for immunologists, flow cytometers can and are used in many different disciplines from basic and applied sciences to diagnostics in biomarker level determination assays.

Flow cytometers can assess many different biological parameters, the basis of which lies in the number of lasers to excite the increasing number of fluorchromes (or mass‐based methods) coupled to antibodies against the desired targets and the ability to accurately measure time‐off flight and dye‐specific fluorescence signals via detectors. In principle and in practice flow cytometry provides a methodology that is relatively easy to understand and learn. This has greatly contributed to the popularity of using flow cytometry as a method of choice, but in the seeming ease of use lies some danger. With the addition of lasers, detectors, filters and especially the development of new dyes, the excitation and emission of which can be overlapping with others in more or lesser degrees, the machine setup, incorporation of controls and data interpretation has become increasingly complex. Within the cytometry community, scientists but especially those that tirelessly work in the flow cytometry core facilities, less than optimal data acquisition and presentation as well as technical issues are frequently spotted. Furthermore, the description of flow cytometry techniques used, gating strategies and controls used in material and method sections of papers is often minimal or even insufficient.

The ARRIVE (Animals in Research: Reporting *In Vivo* Experiments) guidelines 2, were introduced in 2010 with the aim to improve the reporting of the use of animals in biomedical research. More detailed and correct reporting, as simple as reporting the gender and age of animals used, will enhance transparency and reproducibility, thereby enhancing the value of scientific reporting for other academics, industry and policy makers.

We would like to endorse the use of the *European Journal of Immunology* published “Guidelines for the use of flow cytometry and cell sorting in immunological studies” 1 in scientific publications, including manuscripts submitted to *Immunity, Inflammation and Disease*. The guidelines provide an insight into flow cytometry and the technical details, and furthermore, supply an invaluable collection of protocols, hints, advice on controls and instrument setup, trouble shooting on common problems and pitfalls, cautions about technical issues as well as standards for data interpretation. Although the guidelines are not completely settled as they will necessarily evolve, just as science itself does, we urge our authors to critically assess their work in light of these guidelines, to use the guidelines to improve their experimentation where applicable, to discuss the guidelines with their lab members and students and to use the recommendations to improve the reporting of flow cytometry based methods in scientific publications.
